# Musculoskeletal Injuries, Exercise Behaviors, and Reproductive Health Are Related to Physical Fitness of Female First-Responders and Health Care Providers

**DOI:** 10.1089/whr.2023.0189

**Published:** 2024-05-03

**Authors:** C. M. Edwards, J. L. Puranda, É. Miller, M. Aboudlal, N. O’Rourke, M. L. MacDonald, K. B. Adamo

**Affiliations:** School of Human Kinetics, Faculty of Health Sciences, University of Ottawa, Ottawa, Canada.

**Keywords:** postpartum, reproductive health, exercise, occupational health

## Abstract

**Introduction:**

Musculoskeletal injuries (MSKi) are the most common injury type experienced by first-responders and health care providers (HCPs), making them a significant threat to physical and mental well-being. Female reproductive health and injury history has been related to physical fitness in female members of the Canadian Armed Forces. This relationship has not been explored in Canadian protective services personnel (first-responders) or HCPs.

**Methods:**

Fifty-seven females employed as firefighters, paramedics, law enforcements, or HCPs completed a physical fitness protocol to assess the following: (1) muscular power (standing long jump and medicine ball throw), (2) muscular strength (4 repetition maximum (4RM) back squats and bench press), (3) muscular endurance (Biering-Sorenson test, single-leg wall sit, and push-ups), (4) flexibility (sit-and-reach), and (5) aerobic capacity (graded treadmill VO_2max_ test). Spearman *rho* correlation analyses were applied to descriptive analysis, independent-samples *t*-test, one-way ANCOVA (adjusted by age), and chi-square test. Spearman *rho* correlation analyses were used to compare physical fitness results for female reproductive health history (*e.g.,* parity status), previous MSKi, and physical activity behaviors (*e.g.,* sports participation). A *p* value of <0.05 is considered significant.

**Results:**

History of childbirth, body composition, and exercise behaviors were related to physical fitness (*i.e.,* standing long jump, Biering-Sorenson test, bench press, and back squat) in law enforcement, firefighting, paramedicine, and health care personnel.

**Conclusions:**

Physical training programs aimed at supporting parous first-responders or HCPs should emphasize lower body power, lower body strength, and upper body strength.

## Introduction

Musculoskeletal injuries (MSKi) are the most common injury type experienced by first-responders and health care providers (HCPs), making them a significant threat to physical and mental well-being. The occupational injury rate of firefighters is 3–7 times greater than the national average,^1^ police have a 10% greater prevalence of injury than firefighters, (1) and the number of lost-time claims due to injury reported by HCPs was more than double of all other industries (nonmilitary) combined.^2^ While advanced age and service years have been correlated with reduced injury incidence in these occupations, female individuals employed in these roles seem to be at greater risk.^1,3^

Proposed factors contributing to the increased injury risk in females compared with males are muscle activation patterns, anatomical structure, hormone differences, including those associated with the menstrual cycle, and other events associated with female reproduction (*e.g.,* pregnancy and the postpartum).^4^ For example, female military members who have irregular menstrual cycles or have given birth (parous) are more likely to sustain repetitive strain injuries (RSIs).^5,6^ To the best of our knowledge, the relationship between female reproductive health and MSKi has not been explored in Canadian first-responders or health care providers.

When reasoning why parous individuals employed in arduous occupations sustain more MSKi compared with nulliparous peers, physical fitness decline during pregnancy and postpartum must be considered.^7^ Muscular strength, power, endurance, and aerobic capacity have all been associated with injury incidence and prevalence in personnel employed in arduous occupations.^1,8–11^

While physical demands vary between these professions, those employed in these roles benefit from a high fitness level. The day-to-day activities of a paramedic in Canada consists of sedentary periods (*i.e.,* driving) and high physical demand elements such as carrying a 13 kg cardiac monitor or possibly a team lift of stretcher and large patient (200 kg) over a curb.^12^ Low levels of physical fitness can negatively impact job performance and increase the risk of injury.^1,8,13,14^ In policing, higher physical fitness test results are associated with superior marksmanship, including better scores on the decisional shooting test (officers perceive and identify specific targets or threats, distinguishing from nonviable targets).^15^ As the evidence supporting specific physical fitness attributes as protective against injury, such as lower body power or consecutive push-up repetitions, females are severely underrepresented in the literature for individuals employed in arduous occupations.^8,16,17^ The few studies that include females suggest the ability for a physical fitness test ability to predict injury can be sex-dependent.^8,9,16,17^ Research examining optimal exercise methods and physical activity habits supporting occupation performance and injury reduction is equally distorted toward males. This lack of female-specific data highlights a problematic sex-bias when attempting to create evidence-based physical training programming, policy, and procedures.

Ultimately, the presence of females in arduous occupations is increasing and the number of female individuals choosing to continue careers after childbirth is on the rise in Canada.^18^ Research aimed at reducing the burden of MSKi, and understanding the impact of female reproductive health in these occupations is needed. Thus, the purpose of this study is to examine how physical health (reproductive health factors, anthropometric measures, MSKi history) and exercise behavior influence physical fitness in first-responders and HCPs (as defined by the province of Ontario; https://www.ontario.ca/page/regulated-health-professions). We hypothesize that measures of physical fitness will differ by parity status, body composition, and career. We predict outcomes will favor those who are nulliparous, have lower body fat composition, and work as first-responders. Exercise behaviors will also be examined to account for physical adaptations that might occur from strain applied to tissues not in an occupation context.

## Methods

Recruitment was conducted *via* the research laboratory’s social media accounts (*e.g.,* lab website, Facebook, Twitter), the social networks of the researchers, and” snowball” recruiting. The inclusion criteria consisted of the following: (1) biological sex of female, (2) being between the ages of 18 and 55 years, (3) being employed as a health care provider (regulated health professional presently practicing in the province of Ontario [*e.g.,* medical doctor, nurse, kinesiologist, physiotherapist, chiropractor]), firefighter, law enforcement officer, or paramedic, (4) being cleared to engage in maximal exercise (based on the prescreening questionnaire), and (5) providing informed consent to participate (in English or French). The exclusion criteria for this study were as follows: diabetes (any type), untreated thyroid disease, cardiovascular diseases, being pregnant, and contraindication to exercise.

All procedures of the present study have been reviewed and approved by the University Research Ethics Board (H-11–20-6180) and were conducted in accordance with the Declaration of Helsinki.

Data pertaining to female reproductive health (*e.g.,* pregnancy, menstrual cycle, hormonal birth control use), occupation, physical activity and exercise participation, and MSKi history were collected *via* a self-report questionnaire. When available, average daily step counts and physical activity minute data for the year were obtained *via* participant smartwatch history. The following MSKi definitions were provided to participants when self-reporting injury:

*Repetitive Strain or Overuse Injuries—injuries to muscles, tendons, or nerves caused by overuse or repeating the same movement (e.g., ruck marching) over an extended period. For example, carpal tunnel syndrome, tennis elbow, plantar fasciitis, or tendonitis.*

*Acute Injuries—serious physical injuries, likely caused by a significant level of exertion or single incident of trauma, which were serious enough to require at least 24 hours off work after it to recover from. For example, a broken bone, a sprain.*

Participant body weight and composition data were collected using the InBody 520^TM^ (USA) bioelectrical impedance analyzer (BIA). Bone mineral density (BMD) was measured using dual-energy X-ray absorptiometry (DXA). Physical testing was performed to assess the following: (i) muscular power (standing long jump and medicine ball throw), (2) muscular strength (4RM back squats and bench press), (3) muscular endurance (Biering-Sorenson test, single-leg wall sit, and push-ups), (4) flexibility (sit-and-reach), and (5) aerobic capacity (graded treadmill VO_2max_ test) (see Supplementary Appendix A, SDC1, for a detailed description of the protocol).

The physical fitness and body composition protocol used for this study was chosen based on the findings of a scoping review conducted by our laboratory and was used previously with a female military population.^10^ The minimum required sample size for this project was estimated based on an ongoing project occurring in our laboratory, which assessed the relationship between parity status and physical fitness in the Canadian Armed Forces members. Accepting a maximum error of ± 5%, and a desired power of 90%, the minimal sample size was *n* = 48, 24 who have not given birth (nulliparous) and 24 who have given birth (parous).

### Statistical analysis

For MSKi analysis, injuries were stratified by type (acute or RSI), body region, and body complex as follows: (1) head, neck, shoulder, (2) upper extremity (fingers, thumb, hand, wrist, lower arm, elbow upper arm, shoulder), (3) spine (low back, upper back, neck), (4) lumbopelvic hip complex (low back, pelvis, hip), and (5) lower extremity (hip, thigh, knee, lower leg, ankle, foot, toes). Menstrual cycle length and frequency were categorized by (1) regular: within the past year, cycle is consistently between 21–35 days and (2) irregular: within the past year, at least 1 cycle has been outside of 21–35 days (not related to pregnancy). Parity status was defined as (1) parous: has carried at least 1 pregnancy for 20 weeks and (2) nulliparous: has not carried a pregnancy for 20 weeks.

Normality (Shapiro–Wilk) and variance (Levene’s test) assumptions will be used and if non-normally distributed, t-tests will be used to compare the means of continuous descriptive variables as they are considered robust enough for non-normal data.^19^ Significant results from the t-test will then be evaluated for ANCOVA. The bivariate associations for 2 × 2 tables will be analyzed with the chi-square test or Fisher’s exact test (when the assumption of having <20% of the cells with an expected count of less than 5 is not met). Significant findings in the chi-square test will then be assessed using logistic regression analysis when assumptions are met. Numbers and percentages will be used to describe categorical variables. Means and standard deviations will be used to describe continuous variables. Spearman *rho* correlation analyses will also be performed to describe the strength and direction between continuous variables (*e.g.,* anthropometric measures and fitness results). A *p* value <0.05 is considered significant.

## Results

### Participant demographics

A comparison of participant demographics, split by occupation, is presented in Table 1.

**Table 1.** Participant Demographics

Variable	First-responder		Health care provider		*p-*value
	*N*		*N*		
Age (years), mean ± SD	19	34.8 ± 7.4	38	36.9 ± 8.6	0.361
Height (m), mean ± SD	19	167.3 ± 7.7	38	165.9 ± 6.0	0.529
Body weight (kg), mean ± SD	19	73.2 ± 13.3	38	67.3 ± 9.4	0.092
Body fat (%), mean ± SD	19	26.0 ± 9.1	38	28.1 ± 8.7	0.411
BMI (kg/m^2^), mean ± SD	19	26.1 ± 4.1	38	24.2 ± 3.7	0.104
Occupation start (year), mean ± SD	19	2012.7 ± 7.4	38	2012.7 ± 7.5	0.990
Skeletal muscle (%), mean ± SD	19	40.3 ± 6.6	38	39.7 ± 5.5	0.737
Total body BMD (g/cm^2^), mean ± SD	18	1.27 ± 0.111	34	1.21 ± 0.092	0.064
Femur neck BMD (g/cm^2^), mean ± SD	18	1.06 ± 0.113	34	1.02 ± 0.108	0.208
L2–L4 lumbar BMD(g/cm^2^), mean ± SD	18	1.31 ± 0.135	34	1.23 ± 0.140	0.051
1/3^rd^ radius BMD (g/cm^2^), mean ± SD	18	0.720 ± 0.047	34	0.703 ± 0.052	0.231
Parity status					
1 or more births (%)	11	57.9	19	50.0	0.574
Never given birth (%)	8	42.1	19	50.0	
Given birth within 1 year					
Yes (%)	2	10.5	3	7.9	1.000
No (%)	17	89.5	35	92.1	
Menstrual cycle periodicity					
Regular (%)	11	57.9	18	42.1	0.146
Irregular (%)	8	42.1	20	52.6	
Hormone birth control use					
Yes (%)	10	52.6	20	52.6	1.000
No (%)	9	47.4	18	47.4	

*t*-Tests were used to compare means of continuous descriptive variables. Bivariate associations for 2 × 2 tables were analyzed using a chi-square test or Fisher’s exact test (two-sided).

Regular menstrual cycle periodicity = within the past year, cycle is consistently between 21 and 35 days. Irregular menstrual cycle periodicity = within the past year, at least 1 cycle has been outside of 21–35 days. None of the participants has reached menopause.

Significant difference *p* value <0.05.

BMI, body mass index; FR, first-responder; HCPs, health care providers; BMD, bone mineral density.

### Occupation

Nineteen participants were included in the first-responder group and 38 in the health care provider group. Physical fitness comparisons are displayed in Table 2 (significant findings only) (see Supplementary Table Appendix B, SDC 2, for physical fitness results by occupation, parity status, hormone birth control use, menstrual cycle length and frequency).

**Table 2.** Significant Differences in Physical Fitness Test Performance Between First-Responders and Health Care Providers

Fitness metric	First-responders (*n* = 19)	Health care providers (*n* = 38)	*p*-value
Medicine ball toss (cm)	294.7 ± 45.6	244.6 ± 48.7	<0.001^a^
4RM back squat (lbs)	172.63 ± 36.8	207.8 ± 61.8	0.009^a^
4RM bench press (lbs)	110.0 ± 28.3	86.3 ± 20.3	0.003^a^

*t*-Tests (two-sided) were used to compare means of physical fitness test results of participants employed as a first-responder or health care provider.

^a^ Significance set to <0.05.

4RM, 4 repetition maximum.

### Musculoskeletal injuries

Acute injuries were reported by 77.2% of participants, and of the 57 participants included in this study, 78.9% had sustained at least 1 injury within the last 12 months (see Supplementary Table Appendix C, SDC 3, for comparisons of body regions injured by occupation and female reproductive health factors). Note that more first-responders sustained MSKi in the past year (94.7% vs. 71.1%, *p* = 0.045) and RSI overall (94.7% vs. 71.1%, *p* = 0.045) compared with the HCP group. No differences by reproductive health marker for overall RSI, acute injury, or having sustained an injury in the past year.

Participants with a history of back injury performed better on the medicine ball toss test (286.2 cm ± 46.9 vs. 240.4 cm ± 49.1, *p* < 0.001). A history of lumbopelvic hip injury yielded lower times for the Biering-Sorenson test (151.3 seconds ± 48.9 vs. 182.9 ± 67.1, *p* = 0.047). No other injuries, overall or within the last 12-months, were associated with physical fitness (see Supplementary Table Appendix D, SDC 4, for physical fitness results by MSKi stratified by body region).

### Exercise behaviors

Physical activity and exercise participation by occupation are outlined in Table 3. Exercise and physical activity behaviors were associated with fitness test performance and significant relationships are described below (see Supplementary Table Appendix E, SDC 5, for physical fitness results by exercise behavior). Superior long jump was seen in those who participated in team sports (169.9 cm ± 23.5 vs. 152.5 cm ± 24.9, *p* = 0.012). Poorer sit and reach (33.6 cm ± 7.5 vs. 38.3 cm ± 6.9, *p* = 0.028), long jump (145.5 cm ± 18.8 vs. 166.5 ± 25.7, *p* = 0.002), and medicine ball toss (236.7 cm ± 55.4 vs. 274.6 ± 47.2, *p* = 0.004) were observed in participants who reported attending fitness classes virtually. Participants presently following a physical training program created by a fitness professional performed better in the following: (1) sit and reach (40.0 cm ± 5.3 vs. 33.9 cm ± 8.0, *p* < 0.001), (2) absolute weight lifted in the 4RM back squat (203.5 lbs ± 50.6 vs. 167.6 lbs ± 42.2, *p* = 0.005), (3) weight lifted in the 4RM back squat relative to body weight (134.9% ± 33.8 vs. 112.0% ± 29.8, *p* = 0.009), (4) absolute weight lifted in the 4RM bench press (107.2 lbs ± 22.6 vs. 83.7 lbs ± 23.8, *p* <0.001), and (5) weight lifted in the 4RM bench press relative to body weight (71.2% ± 16.5 vs. 56.1% ± 17.2, *p* = 0.002). No relationships were observed between attending fitness classes in person and the test battery.

**Table 3.** Physical Activity and Exercise Participation for First-Responders and Health Care Providers

Variable	*N*	First-responder	*N*	Health care Practitioner	*p*-value
In-person fitness classes					
Yes (%)	8	42.1	13	34.2	0.560
No (%)	11	57.9	25	65.8	
Virtual fitness classes					
Yes (%)	2	10.5	18	47.4	0.008^a^
No (%)	17	89.5	20	52.6	
Follow a PT program made by a fitness professional					
Yes (%)	11	61.1	14	36.8	0.088
No (%)	7	38.9	24	63.2	
Team sports (in the past year)					
Yes (%)	13	68.4	9	24.3	0.001^a^
No (%)	6	31.6	28	75.7	
Nonteam sports, *i.e.,* marathon, cycling, triathlon (in the past year)					
Yes (%)	10	52.6	11	28.9	0.081
No (%)	9	47.4	27	71.1	
Taught or coached how to do a squat previously					
Yes (%)	18	94.7	32	84.2	0.405
No (%)	1	5.3	6	15.8	
Taught or coached how to do a push-up previously					
Yes (%)	16	84.2	27	71.1	0.343
No (%)	3	15.8	11	28.9	
Average daily physical activity minutes, mean ± SD	8	62.5 ± 39.3	17	50.5 ± 25.2	0.447
Average daily step count, mean ± SD	14	8578.5 ± 1790.1	23	9166.9 ± 3249.2	0.483

*t*-Tests were used to compare means of continuous descriptive variables. Bivariate associations for 2 × 2 tables were analyzed using a chi-square test or Fisher’s exact test (two-sided).

^a^ Significant difference *p* value <0.05.

FR, first-responder; HCPs, health care providers; PT, physical training.

### Anthropometrics

Higher skeletal muscle mass percentage was observed in participants who participated in team sports within the past year (42.2% ± 4.9 vs. 38.4 % ± 6.1, *p* = 0.021) and in those who adhere to a physical training program created by a fitness professional (42.3% ± 4.7 vs. 37.9% ± 6.1, *p* = 0.005). Other types of exercise and daily physical activity minutes recorded on smartwatches were not associated with anthropometrics or body composition. However, higher body fat % (*rho =* −0.403, *p* = 0.013) and higher body mass index (BMI) (*rho* = −0.340, *p* = 0.039) were correlated with lower average daily step count for the past year (recorded by smartwatches). To note, having been coached previously on how to perform a push-up or participation in organized team sports (within the past year), and participation in organized nonteam sports (*e.g.,* triathlon, marathon, cycling) was not related to MSKi (acute or RSI).

Body fat percentage, skeletal muscle mass percentage, BMI, height, body weight, and BMD were all correlated with physical fitness test performance (Table 4).

**Table 4.** Correlations Between Anthropometric Measures and Physical Fitness Test Performance of Female First-Responders and Health Care Providers Assessed Using Spearman *Rho* Analysis

Continuous variable			Sit and reach (cm)	Biering-Sorenson (sec)	Bench press 4RM (lbs)	Back squat 4RM (lbs)	Medicine ball toss (cm)	Long jump (cm)	Right leg wall sit (seconds)	Left leg wall sit (seconds)	Push-ups (reps)	VO2max (mL/kg/min)
	Mean ± SD		36.6 ± 7.5	166.8 ± 60.2	94.2 ± 25.6	184.4 ± 49.0	261.3 ± 52.3	159.1 ± 25.4	73.7 ± 43.9	69.5 ± 41.6	24.2 ± 12.1	42.9 ± 7.4
Body fat %	27.0 ± 9.4	Correlation coefficient	−0.215	−0.418^a^	−0.447^a^	−0.230	−0.119	−0.414^a^	−0.378^a^	−0.322^a^	−0.463^a^	−0.675^a^
		*p*-value	0.108	0.001	<0.001	0.085	0.377	0.001	0.004	0.016	<0.001	<0.001
Muscle mass %	39.9 ± 5.9	Correlation coefficient	0.212	0.419^a^	0.471^a^	0.298^a^	0.246	0.512^a^	0.334^a^	0.270^a^	0.422^b^	0.596^a^
		*p*-value	0.114	0.001	<0.001	0.025	0.065	<0.001	0.012	0.044	0.001	<0.001
BMI (kg/m^2^)	24.9 ± 3.9	Correlation coefficient	−0.122	−0.552^a^	-0.070	0.029	0.036	−0.299^a^	−0.432^a^	−0.392^a^	−0.397^a^	−0.581^a^
		*p*-value	0.366	<0.001	0.606	0.830	0.791	0.024	<0.001	0.003	0.002	<0.001
Total body BMD (g/cm^2^)	1.23 ± 0.10	Correlation coefficient	0.134	0.144	0.482^a^	0.444^a^	0.372^a^	0.286^a^	−0.198	−0.193	0.332^a^	0.046
		p-value	0.342	0.308	<0.001	0.001	0.007	0.040	0.163	0.175	0.016	0.758
Femur neck BMD (g/cm^2^)	1.03 ± 0.11	Correlation coefficient	0.008	0.211	0.436^a^	0.457^a^	0.327^a^	0.261	−0.252	−0.247	0.366^b^	0.051
		*p*-value	0.953	0.133	0.001	0.001	0.018	0.062	0.074	0.080	0.008	0.729
L2–L4 BMD (g/cm^2^)	1.26 ± 0.14	Correlation coefficient	0.192	0.025	0.266	0.376^a^	0.374^a^	0.217	−0.317^a^	−0.223	0.106	−0.068
		*p*-value	0.172	0.861	0.057	0.006	0.006	0.122	0.023	0.116	0.453	0.645
1/3^rd^ radius BMD (g/cm^2^)	0.709 ± 0.05	Correlation coefficient	−0.017	−0.108	0.259	0.344^a^	0.150	0.101	−0.230	−0.283^a^	0.106	−0.301^a^
		*p*-value	0.906	0.447	0.064	0.013	0.289	0.477	0.105	0.044	0.455	0.038
Body weight (kg)	69.3 ± 11.1	Correlation coefficient	−0.136	−0.482^a^	0.102	0.121	0.344^a^	−0.129	−0.321^a^	−0.248	−0.303^a^	−0.484^a^
		*p*-value	0.314	<0.001	0.448	0.369	0.009	0.340	0.016	0.065	0.022	<0.001
Height (cm)	166.4 ± 6.6	Correlation coefficient	0.051	−0.04	0.313^a^	0.213	0.526^a^	0.262^a^	0.102	0.166	0.109	0.028
		*p*-value	0.705	0.767	0.018	0.112	<.001	0.049	0.454	0.222	0.418	0.842
2023 average daily step count	8944.3 ± 2773.6	Correlation coefficient	−0.045	0.181	0.412^a^	0.291	0.535^a^	0.286	0.409^a^	0.426^a^	0.427^a^	0.328
		*p*-value	0.792	0.284	0.011	0.08	<0.001	0.086	0.013	0.010	0.008	0.062
2023 average daily physical activity minutes	54.3 ± 30.2	Correlation coefficient	−0.232	−0.059	0.276	0.202	0.440^a^	0.039	0.215	0.346	0.278	0.087
		*p*-value	0.265	0.779	0.181	0.332	0.028	0.855	0.314	0.097	0.179	0.693

Spearman *rho* correlation analysis examining the relationship between (1) body fat percentage and physical fitness test results, (2) muscle mass percentage and physical fitness rest results, and (3) daily activity habits.

Note that due to limitation of smartwatch use, daily physical activity *n* = 25 and daily step count *n* = 37.

^a^ Significant difference <0.05 (2-tailed).

BMD, bone mineral density; BMI, body mass index.

### Reproductive health

Two-sided *t*-tests yielded parity status as a predictor for muscular power and strength, results are outlined in Table 5 (significant findings only) (see Supplementary Table Appendix B, SDC 1, for physical fitness results by occupation, parity status, hormone birth control use, menstrual cycle length and frequency). The relationships between the covariates age and body fat % with parity status violated assumption checks for the ANCOVA, and therefore, adjusted analyses were not performed. To note, having given birth within the past year (*n* = 5) was not related to physical fitness performance.

**Table 5.** A Comparison of Physical Fitness Test Results Between Nulliparous and Parous First-Responders and Health Care Providers

Fitness metric	Nulliparous (*n* = 27)	Parous (*n* = 30)	Significance
Long jump (cm)	167.4 ± 26.8	151.6 ± 21.9	0.017^a^
4RM back squat (R%)	133.0 ± 35.8	113.3 ± 28.3	0.024^a^
4RM bench press (R%)	68.3 ± 21.1	57.9 ± 13.7	0.029^a^

*t*-Tests (two-sided) were used to compare means of physical fitness test results of nulliparous and parous participants.

^a^ Significance set to <0.05.

RM, repetition maximum; R%, bodyweight/absolute weight lifted in RM.

A normal (21–35 days) and regular menstrual cycle length for the last 12 months was reported by 50.9% of participants and was not a predictor of physical fitness performance. To note, almost half (48.2%) of participants reported using an app or calendar to track menstrual cycle symptoms. Hormone birth control use was reported by 52.6% of participants with no relationship with physical fitness performance.

## Discussion

To our knowledge, this study is the first comprehensive investigation into reproductive health, MSKi, and body composition as predictors of physical fitness in female first-responders and HCPs in Canada. Supporting our hypothesis, this study contributes that occupation, body composition, previous MSKi, and a history of childbirth influence physical fitness in female individuals employed in arduous occupations.

### Anthropometrics

Our findings indicate that higher body weight is correlated with decreased aerobic capacity, upper body power, and full body muscular endurance in both occupational groups. Furthermore, BMI, body fat percentage, skeletal muscle mass percentage, and BMD were also related to physical fitness performance. These results are consistent with present literature comparing the body composition of servicewomen with military task performance and fitness testing. Research linking decreased aerobic capacity, as well as poorer upper and lower body strength, with higher relative body fat percentage and lower muscle mass is presently used to guide the body composition standards of the U.S. Marine Corps.^20,21^ Similar to what is observed in the elite female warfighters,^22^ our data indicate that a higher BMD is associated with increased physical performance. The literature describing the relationship between body composition and fitness of female first-responders and HCPs in Canada is limited. Our study therefore confirms the importance of increased skeletal muscle mass, decreased body fat percentage, and decreased body weight in females working in arduous occupations within Canada.

### Reproductive health

While no significant differences were observed in body weight, body composition, or BMD between parous and nulliparous groups, parity status was related to fitness performance. Nulliparous participants achieved better results in a number of the tests conducted in the present study, supporting our hypothesis. For example, participants in the present study who had not given birth previously were able to jump significantly further compared with the parous group (167.4 cm ± 26.8 vs. 151.6 ± 21.6, *p* = 0.017). Given that childbirth results in significant structural changes to the pelvic floor, increasing the risk of urinary incontinence and pelvic floor dysfunction, the parous group may avoid high-impact exercises (*i.e.,* jumping) in their training programs or chosen fitness regimen.^23^ Jump performance could also be influenced by the higher rate of foot injuries reported by the parous individuals in our sample compared with the nulliparous group (*p* = 0.027). This body region has previously been identified as more vulnerable in parous individuals. A recent study by our group/team utilizing an online questionnaire collected information from 748 female members of the Canadian Armed Forces concluded that participants with a history of childbirth were more likely to have experienced a foot RSI (parous = 39.3% vs. nulliparous = 24.1%, aOR: 1.79, CI: 1.24; 2.59) during their career.^5^ The higher risk of injury at this body region is likely due to increases in body mass and endocrine system responses to pregnancy resulting in anatomical changes in the foot (*i.e.,* increased length, width, and volume).^24–27^ As body mass may be contributing to injuries in the foot and body composition was related to physical performance, these factors may be interacting to impair lower body power.

The difference in jump performance may also relate to lower body strength, as demonstrated by the back squat in our protocol, jump ability was correlated with back squat strength (*rho =* 0.524, *p* > 0.001).^28^ While heavy lifting during pregnancy has been discouraged previously, newly published findings contradict this theory. Prevett et al. (2023) demonstrate that heavy resistance training throughout pregnancy and postpartum, lifting ≥80% of 1 RM during pregnancy, can be safe.^29^ The aforementioned study also found that maintaining prepregnancy training levels until delivery may reduce reproductive complications. Presently, it is common practice to decrease physical training load and volume during pregnancy and the postpartum period. The decline or cessation of heavy resistance training might place parous individuals at a disadvantage compared with their nulliparous peers who continue to maintain strength adaptations. This theory could also explain the significantly higher bench press performance in the nulliparous group. Physical training should target this discrepancy as upper body strength can be important for the safety of first-responders and HCPs during a number of occupation-related tasks, such as equipment handling, lifting patients, or if required to restrain uncooperative individuals. Parity status, expanding beyond the postpartum period, as a predictor for physical fitness had not been explored previously in Canadian first-responders or HCPs. Factors such as parental leave policies, professional physical training support, type of birth, or number of children could be contributing to the diminished fitness performance. Our findings justify initiatives and investigations aimed specifically at supporting females who bear children and choose to return to physically demanding careers.

### Physical activity and exercise behaviors

Team sport participation is a valuable platform for first-responders to learn occupation-related skills and ideals encourage social connection, while improving physical fitness.^30^ Research conducted on Canadian military personnel indicate that sport participation can also improve mental health status, a benefit that must be considered when supporting a population with high rates of suicide.^31^ Given that a recent systematic review identified that 8.9%–74.2% of HCPs experience symptoms of depression, with females being at greater risk,^32^ the combined mental and physical health benefits of sport participation make this an appropriate exercise modality for both first-responders and HCPs. Our findings indicate that first-responders were more likely to have participated in team sports within the past year than the HCP group. Participation in team sports was related to better standing long jump performance, and lower jump distance was associated with injury prevalence. HCPs in our sample were more drawn to virtual fitness classes. Limited comparative data for fitness benefits yielded from virtual compared with in-person physical training classes exist, with only one study identified when reviewing the literature. The aforementioned publication that compared the effects of a 10-week high-intensity interval training program for obese and overweight female individuals, delivered in-person (*n* = 9) and virtually (*n* = 11), demonstrated better fitness results in the on-site group.^33^ Although physical and physiological adaptations can certainly be accrued from virtual class participation,^33^ other forms of exercise (*i.e.,* team sports, follow a PT program, or attend in-person fitness classes) appear to yield greater physical fitness benefits. Our results showing better flexibility, stronger lower body, and higher upper body strength indicate that subscribing to a physical training program created by a fitness professional may be the optimal choice for achieving higher levels of fitness.

Physical training created and administered by qualified professionals improves injury rehabilitation results,^34^ and the value of trainer qualifications on physical fitness outcomes has been demonstrated previously.^35^ Roos et al. (2015) found that ten weeks of physical training designed and administered by a coach with a degree in physical education, compared with a programming by army physical training instructors, yield superior strength, balance, and aerobic endurance.^35^ Seemingly, participants in our sample seek professional support after sustaining injury. While 44.6% of the participants in our study presently follow a training program made by a fitness professional (*e.g.,* strength and conditioning coach, kinesiologist, physical trainer), 96.0% of those in this group had sustained an RSI previously (*p* = 0.004). This compares with 44.4% of participants who attend fitness classes in person (*e.g.,* spin, yoga, high-intensity interval training) having RSI history (*p* = 0.021). As health care professionals and first-responders rely on subject matter experts and often collaborate with specialists, it is possible that individuals in these occupations apply a similar approach to their own health. For example, they may opt to utilize individualized professional support for physical training following an injury to avoid exacerbating a previous injury.

Based on our findings and the present literature, investigations and interventions involving the effectiveness of exercise and physical training should consider delivery method, qualifications of the individual administering the program, and differentiate between team and nonteam sports. While the focus of this study was to explore factors contributing to physical fitness in females employed as first-responders or in health care, psychological strain on individuals in these occupations must be acknowledged. Future research examining the impact of various physical training modality participations on burnout and other aspects mental health, in addition to investigations of the relationship between psychological health and physical fitness would be helpful for guiding health initiatives for these individuals.

### Limitations

This analytical cross-sectional study combines physiological testing with a retrospective component (*i.e.,* questionnaire). The risk of recall bias is increased due to the injury data being gathered by a self-report questionnaire. Injury data collected *via* self-report have been validated in arduous occupations, although underreporting *via* this method has also been demonstrated.^36^ While the sample size included in the present study was beyond the initial power calculation performed for the project, the statistical analyses used were limited based on the number of participants in some categories. Thus, where small sample sizes were compared, findings should be considered preliminary. A larger sample would be especially advantageous for the injury and occupation comparisons, as it would enable the inclusion of covariates and reduce the risk of chance relationships. The effect of age could be especially insightful to assess, as peak strength in females is seen between 30 and 39 years of age, runners who plan to return to sport after pregnancy have no statistical decrease in performance 1–3 years postpregnancy and over half have significant performance improvements, and there is a considerable literature gap examining the relationship of reproductive health status through the female life span.^32,37^ A larger sample would also permit for a more robust assessment of reproductive health variables, such as differentiating between oligomenorrhea and amenorrhea or performing postpartum and parity status comparisons. To note, a requirement for participation in the present study was the ability to attend testing at the University of Ottawa, participants were all members of municipal and provincial organizations based in the National Capital Region of Canada. The pregnancy policies, maternity benefits, and return-to-work requirements vary between these organizations limiting job-specific recommendations from our findings. Another limitation may also be the lack of smartwatch standardization. While the data collected by smartwatches can provide valuable insight into daily physical activity minutes or step counts, validity between devices varies. The physical activity data included in this study should therefore be viewed as approximations. This study was not designed to determine causality; further investigation is needed to understand the relationships observed.

## Conclusion

MSKi are prevalent among females in arduous occupations overall, but were not overwhelmingly predictive of physical fitness. Conversely, a history of childbirth, body composition, and exercise behaviors were related to physical fitness in law enforcement, firefighting, paramedicine, and health care personnel. The minimal interaction between MSKi history and physical fitness may be due, in part, to physical training practices. Thus, interventions for females employed in arduous roles should consider exercise type and delivery method based on specific occupation category. Physical fitness programs aimed at supporting parous first-responders or HCPs should emphasize lower body power, lower body strength, and upper body strength. Furthermore, the needs analysis for physical training and physical therapy program design should be informed by occupation, reproductive health factors, injury history, and body composition.

## Supplementary Material

Supplementary Appendix A

Supplementary Appendix B

Supplementary Appendix C

Supplementary Appendix D

Supplementary Appendix E

## Abbreviation Used

ANCOVA: Analysis of variance
BIA: Bioelectrical impedance analyzer
BMD: Bone mineral density
BMI: Body mass index
CAF: Canadian Armed Forces
DXA: Dual-entry X-ray absorptiometry
FR: First-responders
HCP: Healthcare provider
MSKi: Musculoskeletal injury
PT: Physical training
RM: Repetition maximum
RSI: Repetitive strain injury
SD: Standard deviation
US: United State
USA: United States of America

## References

[1] Lentz L, Randall JR, Gross DP, et al. The relationship between physical fitness and occupational injury in emergency responders: A systematic review. Am J Ind Med 2019;62(1):3–13; doi: 10.1002/ajim.22929 30548649

[2] Canada AoWCBo. National Work Injury, Disease and Fatality Statistics. In: Canada NAICS., editor. 2022. Available from: https://awcbc.org/en/statistics/ [Last accessed: August 22, 2023].

[3] Poplin GS, Roe DJ, Peate W, et al. The association of aerobic fitness with injuries in the fire service. Am J Epidemiol 2014;179(2):149–155; doi: 10.1093/aje/kwt213 24186973

[4] Wolf JM, Cannada L, Van Heest AE, et al. Male and female differences in musculoskeletal disease. J Am Acad Orthop Surg 2015;23(6):339–347; doi: 10.5435/JAAOS-D-14-00020 26001426

[5] Edwards CM, Miller E, da Silva DF, et al. Does a history of childbirth impact injury prevalence and mental health in female military members? Appl Physiol Nutr Metab 2023;48(11):841–850; doi: 10.1139/apnm-2023-0028 37429041

[6] Puranda JL, Silva D, Edwards CM, et al. Association between reproductive health factors and musculoskeletal injuries in female canadian armed forces members. J Womens Health (Larchmt) 2022;32(2):199–207; doi: 10.1089/jwh.2021.0647 36094835

[7] Iobst SE, Smith DC, Best NI, et al. A scoping review of pregnancy, childbirth, and the postpartum period in active duty U.S. Military women. Womens Health Issues 2021;31 Suppl 1:S81–S92; doi: 10.1016/j.whi.2020.05.005 34454706

[8] Lentz L, Randall JR, Guptill CA, et al. The association between fitness test scores and musculoskeletal injury in police officers. Int J Environ Res Public Health 2019;16(23):4667; doi: 10.3390/ijerph16234667 31771132 PMC6926534

[9] de la Motte SJ, Lisman P, Gribbin TC, et al. Systematic review of the association between physical fitness and musculoskeletal injury risk: part 3—flexibility, power, speed, balance, and agility. J Strength Cond Res 2019;33(6):1723–1735; doi: 10.1519/JSC.0000000000002382 29239989

[10] Barbeau P, Michaud A, Hamel C, et al. Musculoskeletal injuries among females in the military: A scoping review. Mil Med 2021;186(9–10):e903–e931; doi: 10.1093/milmed/usaa555 33367692

[11] Carr-Pries NJ, Killip SC, MacDermid JC. Scoping review of the occurrence and characteristics of firefighter exercise and training injuries. Int Arch Occup Environ Health 2022;95(5):909–925; doi: 10.1007/s00420-022-01847-7 35266040

[12] Coffey B, MacPhee R, Socha D, et al. A physical demands description of paramedic work in Canada. Int J Industrial Ergonomics 2016;53:355–362; doi: 10.1016/j.ergon.2016.04.005

[13] Nindl BC, Jones BH, Van Arsdale SJ, et al. Operational physical performance and fitness in military women: Physiological, musculoskeletal injury, and optimized physical training considerations for successfully integrating women into combat-centric military occupations. Mil Med 2016;181(1 Suppl):50–62; doi: 10.7205/MILMED-D-15-00382 26741902

[14] Siddall AG, Stevenson RDM, Turner PJF, et al. Physical and physiological performance determinants of a firefighting simulation test. J Occup Environ Med 2018;60(7):637–643; doi: 10.1097/JOM.0000000000001313 29485491

[15] Muirhead H, Orr R, Schram B, et al. The relationship between fitness and marksmanship in police officers. Safety 2019;5(3):54; doi: 10.3390/safety5030054

[16] Lisman PJ, de la Motte SJ, Gribbin TC, et al. A systematic review of the association between physical fitness and musculoskeletal injury risk: part 1—cardiorespiratory endurance. J Strength Cond Res 2017;31(6):1744–1757; doi: 10.1519/JSC.0000000000001855 28538328

[17] de la Motte SJ, Gribbin TC, Lisman P, et al. Systematic review of the association between physical fitness and musculoskeletal injury risk: part 2—muscular endurance and muscular strength. J Strength Cond Res 2017;31(11):3218–3234; doi: 10.1519/JSC.0000000000002174 28796127

[18] Choi Y. The likelihood and timing of mothers returning to work after parental leave. Economic and Social Reports 2023. Available from: https://policycommons.net/artifacts/4250682/the-likelihood-and-timing-of-mothers-returning-to-work-after-parental-leave-by-youjin-choi/5059821/ [Last accessed: August 21, 2023].

[19] Le Cessie S, Goeman JJ, Dekkers OM. Who is afraid of non-normal data? Choosing between parametric and non-parametric tests. Eur J Endocrinol 2020;182(2):E1–E3; doi: 10.1530/EJE-19-0922 31910149

[20] Allison KF, Keenan KA, Lovalekar M, et al. Fight load index and body composition are most associated with combat fitness in female Marines. J Sci Med Sport 2019;22(4):494–499; doi: 10.1016/j.jsams.2018.10.014 30448087

[21] Crawford K, Fleishman K, Abt JP, et al. Less body fat improves physical and physiological performance in army soldiers. Mil Med 2011;176(1):35–43; doi: 10.7205/MILMED-D-10-00003 21305957

[22] Mcclung H, Spiering BA, Bartlett PM, et al. Physical and Physiological Characterization of Female Elite Warfighters. Med Sci Sports Exerc 2022;54(9):1527–1533; doi: 10.1249/MSS.0000000000002942 35621397 PMC9390221

[23] Puranda JL, da Silva DF, Edwards CM, et al. Characteristics Associated with Pelvic Floor Disorders among Female Canadian Armed Forces Members. J Obstet Gynaecol Can 2023;45(9):646–654; doi: 10.1016/j.jogc.2023.05.027 37268158

[24] Gimunová M, Zvonař M, Sebera M, et al. Special footwear designed for pregnant women and its effect on kinematic gait parameters during pregnancy and postpartum period. PLoS One 2020;15(5):e0232901-e; doi: 10.1371/journal.pone.0232901 32396578 PMC7217473

[25] Wetz H, Hentschel J, Drerup B, et al. Changes in shape and size of the foot during pregnancy. Orthopade 2006;35(11):1124, 1126–30; doi: 10.1007/s00132-006-1011-1 17061079

[26] Vico Pardo FJ, López del Amo A, Pardo Rios M, et al. Changes in foot posture during pregnancy and their relation with musculoskeletal pain: A longitudinal cohort study. Women Birth 2018;31(2):e84–e8; doi: 10.1016/j.wombi.2017.08.114 28888865

[27] Christopher SM, Bauer L, Maylone R, et al. Biomechanical and musculoskeletal differences between postpartum runners and nulliparous controls. J Women's Health Phys Ther 2022;46(1):11–17; doi: 10.1097/JWH.0000000000000226

[28] Haun CT, Martin JS, Gleason BH, et al. Static jump test performance is related to back squat strength in athletes. International Journal of Sports Science & Coaching 2017;12(5):653–660; doi: 10.1177/1747954117727843

[29] Prevett C, Kimber ML, Forner L, et al. Impact of heavy resistance training on pregnancy and postpartum health outcomes. Int Urogynecol J 2023;34(2):405–411; doi: 10.1007/s00192-022-05393-1 36331580

[30] Lindberg O, Rantatalo O, Stenling C. Police bodies and police minds: Professional learning through bodily practices of sport participation. Studies in Continuing Education 2017;39(3):371–387; doi: 10.1080/0158037X.2017.1337631

[31] Kinoshita K, MacIntosh E, Sato S. Association of latent mental health profiles of military officers with active sport participation and hedonic and eudaimonic motives for sport participation. Sport, Exercise, and Performance Psychology 2022;11(3):353–368; doi: 10.1037/spy0000291

[32] Darroch F, Schneeberg A, Brodie R, et al. Effect of pregnancy in 42 elite to world-class runners on training and performance outcomes. Med Sci Sports Exerc 2023;55(1):93–100; doi: 10.1249/mss.0000000000003025 35975937

[33] Zito GC. Comparison of an Onsite vs Virtual Fitness Program on Anthropometric Measures, Aerobic Fitness, and Vascular Markers of Cardiac Risk in Overweight/Obese Premenopausal Women, University of Miami; 2022. Available from: https://na-st01.ext.exlibrisgroup.com/01UOML_INST/upload/1711740933965/gcz5Sp22.pdf?Expires=1711741054&Signature=Q8mucwnR56mqDE-7XEXY6FLIFrx6rzWei-fLHTe9qxKLA9k5-6U3WWuOR8zIBfmydCM2Y1d∼Pi7WZjPm3RIBVUjZoDCHh0ldDZGAeANlztjJSOujVUShTxj5A3TTr∼P5tBxqOOI6Aj-GL1JEmb1PTC9fVNSpUBgTLCkdl-DdPjo0ODlxxWRmSUr3Vkhf2B3ovh26VHNUytWvk54pUPiWQqB7kcUtWPy0jzLnCcjMaHktES7whcZxOPk-28KpMoO5IAdMoTNpxXtJI9J8PGe65J3mkUw∼Aotbsn-∼KMvgcqWw0s8TsA∼eFTCv1l6v5QtP–XXDGHOxitkMA9eBbVyZg__&Key-Pair-Id=APKAJ72OZCZ36VGVASIA [Last accessed August 2, 2023].

[34] Cox L, Kohne H, Schwieterman A, et al. Effects of a Functional Bridge Program on Physical Performance and Injury Status of Military Population at Risk for Musculoskeletal Injury. Air Force Research Laboratory: States Air Force; 2020. Contract No.: AD1117615. Available from: https://apps.dtic.mil/sti/citations/AD1117615 [Last accessed August 2, 2023].

[35] Roos L, Hofstetter M-C, Mäder U, et al. Training Methods and Training Instructors' Qualification Are Related to Recruits' Fitness Development During Basic Military Training. J Strength Cond Res 2015;29 Suppl 11:S178–S186. 26506185 10.1519/JSC.0000000000001106

[36] Schuh-Renner A, Canham-Chervak M, Grier TL, et al. Accuracy of self-reported injuries compared to medical record data. Musculoskelet Sci Pract 2019;39:39–44; doi: 10.1016/j.msksp.2018.11.007 30472439

[37] Perna FM, Coa K, Troiano RP, et al. Muscular grip strength estimates of the U.S. Population from the National Health and Nutrition Examination Survey 2011-2012. J Strength Cond Res 2016;30(3):867–874; doi: 10.1519/JSC.0000000000001104 26196662 PMC7197498

