# Supplementary material for: Musculoskeletal Injuries, Exercise Behaviors, and Reproductive Health Are Related to Physical Fitness of Female First-Responders and Health Care Providers
Source: Womens Health Rep (New Rochelle). 2024 May 3;5(1):393–403. doi: 10.1089/whr.2023.0189 (PMC11257141; doi:10.1089/whr.2023.0189)
Supplement: Supplementary Appendix A [file whr.2023.0189_suppl_appa.docx]

SDC 1.

Appendix A. Anthropometric measures and physical fitness assessment protocol

i) **Body composition:**

Participants were asked to further remove any metal objects on their person (i.e., watch, rings, other jewelry). Individuals then stood on the InBody® (USA) bioelectrical impedance analyzer (BIA), hold the body composition analyzer arms. The BIA device directed the person on how long they needed to remain standing or if they needed to reposition. Body weight was also obtained during this test. This test was performed fasted (8 hours nothing to eat or drink).

ii) **Bone mineral density:**

*DXA measurement*: The DXA device used was GE Lunar Prodigy (GE Healthcare Lunar, Madison, MI, USA). Participants wore fitted clothing and removed all metals from their person. Participants were fasted (nothing to eat or drink for a minimum of 8 hours prior to attending study) and were asked not to engage in strenuous physical activity in the last 24hrs. Four scans took place: i) Full body, participants lay on their back on the device, ii) Right forearm, participants sat in an upright position beside the DXA with their arm on the device and secured to a board by two straps, iii) Lumbar spine, participants lay in a supine position with their legs place on a positioner box secured by straps at the knees and ankles, iv) Femur, participants lay in a supine position with their legs place on a positioner box secured by straps at the knees and ankles. Participants were positioned by a member of the research team. The DXA operator received DXA training from a representative of GE Healthcare.

After the bone mineral density test, participants were permitted to eat and drink.

A standardized warm-up was performed. The standardized warm-up started general and ended with movements specific to the test protocol. The general phase (2 min): jogging, high-knees, high-heels, walking lunges. General to specific phase (4 min): static and dynamic stretching and activation. Specific phase (4 min): body weight/low weight repetitions of the movements included in the test. During this phase, movement coaching occurred for the maximal strength tests.

iii) **Flexibility**: The sit-and-reach test box was be placed against a wall. With their shoes off, participants sat with the bottom of their feet against the box and their legs extended. With one hand placed over the other (palm side down), their arms outstretched parallel to the floor and directed to their toes, participants leaned forward in a slow and steady movement to briefly hold and achieve the greatest distance. Participants performed 3 attempts with a 30-second rest interval between each repetition.

iv) **Muscular power**: standing long jump and medicine ball explosive power tests was performed to assess lower- or upper-body power. For the jump tests, each subject stood at the starting position with their legs parallel and feet shoulder-width apart. Participants were instructed to bend at the knees and bring the arms behind the body. Then, with a powerful drive, they extended their legs, thrust their arms forward and jump as far horizontally as possible (standing long jump). For the standing long jump, individuals were asked to ‘hold’ the landing while the distance was measured from the start line to the back of the closest heel.

For the medicine ball explosive power test, subjects sat on a chair placed against a wall, holding a medicine ball. A tape measure was placed on the ground starting at the front end of the chair of the subject and stretched out to a distance of 15 m. With their backs against the chair for support and their feet flat on the ground, participants were advised to ‘push’ a 3 kg medicine ball, originally held at the level of the centre of their chest, away as far as possible using a motion similar to a basketball chest pass.

The best of three attempts was recorded for each power test and a 1 min rest interval allowed between repetitions.

v) **Strength**: Four repetition maximum (RM) were performed in bench press and back squat exercises. Participants had 7 attempts for each exercise with 3-5min interval between them. Weight progression recommendations were 5 kg for bench press, and 10 kg for the back squat however participants could request different progression weights. Two or three spotters followed all the strength tests to ensure safety of the participant (one on each side of the bar and one behind during the squats). 5-10 minutes rest between the back squat and bench press tests were provided to the participants.

vi) **Endurance strength**: Push-up test (maximum number of repetitions), Biering-Sorensen test (time to exhaustion), and single leg wall sit (time for each leg with 1-min interval between legs) to assess muscle edurance in both upper- and lower-body. There was a 20 minute interval between endurance strength tests and the aerobic fitness test.

vii) **Aerobic fitness**: A graded treadmill exercise test (GXT) based on Modified Balke protocol and indirect calorimetry individual’s maximal aerobic capacity (VO_2max_) was applied to measure the gold-standard maximal oxygen uptake (VO_2max_). Expired gases was collected and analyzed using the wearable and portable metabolic system (K5, COSMED s.r.l., Rome, Italy). A Hans Rudolph (Hans Rudolph Inc, Kansas, USA) facemask and head support will be fitted on the participant and attached to the K5 unit. A Polar Wear Link and coded transmitter (Polar Electro Canada Inc, Lachine, QC) to monitor heart rate was fitted and secured around the chest beneath the participant’s nipple line, next to the skin. The Polar H800 receiver (watch) was be secured to the treadmill and monitored by the assessors.
Prior to the start of the test, participants had their resting HR measured and participants were permitted to familiarize themselves with treadmill running by warming up for a period of 5 minutes. Age-predicted heart rate reserve is determined using the Karvonen method for calculating reserve: HR_max_-HR_rest_. While there is debate regarding accurate determination of age-predicted HRmax, when combined with HR_reserve_ it can be useful in establishing an evidence- based running speed for the GXT. HR_max_ is determined using the following equation: 208-0.7*age.

*Warm-up*: Participants did not wear to the breathing apparatus (i.e., facemask and head support) for the warm-up, but did wear the Polar heart rate (HR) monitor. Participants started running on the treadmill at a speed of 4.0 miles per hour (mph) and speed gradually increased until they reach a comfortable warm-up speed between 5.0 – 7.0 mph. The treadmill speed for the test was based on the speed at which the participant attained a HR greater than 75% of their age-predicted heart rate reserve during the warm-up. Immediately after the determination of initial running speed for the test, the participant was be fitted to the breathing apparatus (i.e., facemask and head support). Participants stood for 3-5 minute in a standing position to collect baseline metabolic values while allowing one to get accustomed to wearing and breathing through the mask. This standing period allowed us to ensure the values being collected were valid prior to the actual test.

*Test*: Using the speed determined during the warm-up, the first 2 min of the treadmill test were completed at a grade of 0%. After that, the treadmill incline increased by 2% every 2 min until a respiratory exchange ratio (RER) of 1.0 was achieved. When an RER value of 1.0 was achieved, the treadmill incline was be increased by 1% every minute until volitional fatigue, at which time the test was be terminated. At the end of each 2-min test increment, up to when an RER value of 1.0 was achieved, the participants provided a rating of their perceived exertion using a Borg Scale, as rate of perceived exertion (RPE) has been found to be a valuable and reliable indicator in monitoring an individual’s exercise tolerance. RPE correlates highly with measured exercise HR and were developed to allow the exerciser to subjectively rate their feelings during exercise.
Every effort was made to conduct the test in a manner to minimize discomfort and risk. The participant was notified that they may stop the test at any time. Criteria for the termination of the VO_2max_ test included any of the following:
• onset of angina (chest pain) or angina-like symptoms
• signs of poor perfusion – light headedness, confusion, pallor (pale appearance to the skin), cyanosis (bluish discoloration); ataxia (failure of muscular coordination), nausea, or cold and clammy skin
• participant requested to stop
• volitional fatigue
• physical or verbal manifestations of severe fatigue
• failure of testing equipment
• shortness of breath, wheezing, leg cramps
• failure of HR to increase with increased exercise intensity
Any participant that displays one or a combination of any of the previously mentioned criteria for test termination was to be referred to the appropriate medical authorities on-site. Various objective and subjective indicators are useful to confirm that maximal effort has been elicited during the GXT. The following indicators were be used to confirm VO_2max_:
• failure of HR to increase with further increases in exercise intensity
• a plateau in oxygen uptake (or failure to increase oxygen uptake by 150 ml/min) with increased workload
• an RER greater than 1.15
• an RPE of more than 17 (6 to 20 scale)
