# Supplementary material for: Musculoskeletal Injuries, Exercise Behaviors, and Reproductive Health Are Related to Physical Fitness of Female First-Responders and Health Care Providers
Source: Womens Health Rep (New Rochelle). 2024 May 3;5(1):393–403. doi: 10.1089/whr.2023.0189 (PMC11257141; doi:10.1089/whr.2023.0189)
Supplement: Supplementary Appendix B [file whr.2023.0189_suppl_appb.docx]

|  | Occupation | | | | Parity Status | | | | Hormone Birth Control | | | | Menstrual cycle length and frequency | | | |
| --- | --- | --- | --- | --- | --- | --- | --- | --- | --- | --- | --- | --- | --- | --- | --- | --- |
| Fitness Metric | First-responders  (n = 19) | HCP  (n = 38) | Significance | Nulliparous  (n = 27) | | Parous  (n = 30) | Significance | Yes %  (n = 30) | | No %  (n = 27) | Significance | Regular  (n = 29) | | Irregular  (n = 28) | Significance |  |
| Sit-and-Reach (cm) | 36.9± 6.7 | 36.5 ± 7.9 | 0.860 | 37.8 ± 7.4 | | 35.6 ± 7.5 | 0.273 | 37.5 ± 7.5 | | 35.8 ± 7.5 | 0.405 | 35.8 ± 7.8 | | 37.5 ± 7.2 | 0.389 |  |
| Long Jump (cm) | 167.5 ± 22.8 | 154.9 ± 25.9 | 0.066 | 167.4 ± 26.8 | | 151.6 ± 21.9 | 0.017* | 164.6 ± 25.8 | | 153.0 ± 23.9 | 0.085 | 160.8 ± 25.6 | | 157.3 ± 25.5 | 0.616 |  |
| Medicine Ball Toss (cm) | 294.7 ± 45.6 | 244.6 ± 48.7 | <0.001* | 265.2 ± 46.1 | | 257.7 ± 59.1 | 0.599 | 276.9 ± 51.1 | | 253.9 ± 54.9 | 0.321 | 262.1 ± 50.6 | | 262.1 ± 56.2 | 0.907 |  |
| 4RM Back Squat (R%) | 131.8 ± 41.6 | 118.1 ± 27.7 | 0.142 | 133.0 ± 35.8 | | 113.3 ± 28.3 | 0.024* | 124.2% ± 30.4% | | 120.9% ± 36.7% | 0.715 | 130.2% ± 28.1% | | 114.8% ± 36.7% | 0.080 |  |
| 4RM Back Squat (lbs) | 172.63 ± 36.8 | 207.8 ± 61.8 | 0.009* | 194.9 ± 50.9 | | 174.8 ± 46.1 | 0.123 | 189.3 ± 42.4 | | 178.8 ± 55.7 | 0.423 | 191.0 ± 44.0 | | 177.4 ± 53.7 | 0.299 |  |
| 4RM Bench Press (R%) | 70.0 ± 20.2 | 59.2 ± 16.3 | 0.054 | 68.3 ± 21.1 | | 57.9 ± 13.7 | 0.029* | 61.9% ± 17.4% | | 63.8% ± 19.4% | 0.703 | 66.5% ± 17.1% | | 59.0% ± 18.8% | 0.119 |  |
| 4RM Bench Press (lbs) | 110.0 ± 28.3 | 86.3 ± 20.3 | 0.003* | 99.8 ± 29.7 | | 89.2 ± 20.6 | 0.118 | 94.7 ± 25.7 | | 93.7 ± 26.1 | 0.889 | 96.9 ± 22.6 | | 91.4 ± 28.6 | 0.426 |  |
| Biering Sorenson (sec) | 153. 2 ± 53.0 | 173.6 ± 63.1 | 0.206 | 165.7 ± 56.7 | | 167.8 ± 64.1 | 0.893 | 168.4 ± 53.1 | | 165.1 ± 68.3 | 0.841 | 174.1 ± 61.0 | | 159.3 ± 59.5 | 0.357 |  |
| Single-leg Wall Sit (Rt) | 78.2 ± 54.9 | 71.6 ± 38.2 | 0.652 | 73.2 ± 46.6 | | 74.2 ± 42.1 | 0.934 | 67.1± 42.0 | | 81.4 ± 45.5 | 0.228 | 82.0 ± 39.9 | | 64.9± 46.8 | 0.145 |  |
| Single-leg Wall Sit (L) | 68.9 ± 44.2 | 70.8 ± 36.6 | 0.869 | 64.8 ± 31.7 | | 73.6 ± 48.8 | 0.432 | 68.6± 49.5 | | 70.5 ± 31.1 | 0.869 | 79.6 ± 44.2 | | 58.7 ± 36.4 | 0.059 |  |
| Push-ups (reps) | 27.7 ± 13.6 | 22.4 ± 11.0 | 0.150 | 26.5 ± 13.1 | | 22.1 ± 11 | 0.178 | 25.0 ± 11.8 | | 23.3 ± 12.6 | 0.585 | 26.8 ± 11.3 | | 21.5 ± 12.5 | 0.094 |  |
| VO_2max_(mL/kg/min) | 43.6 ± 8.5 | 42.6 ± 7.0 | 0.687 | 44.6 ± 7.0 | | 41.3± 7.6 | 0.113 | 42.6 ± 7.3 | | 43.2 ± 7.8 | 0.785 | 43.6 ± 6.9 | | 42.2 ± 8.0 | 0.496 |  |

SDC 2.

Appendix B: Physical fitness results by occupation, parity status, hormone birth control use, menstrual cycle length and frequency

Note: T-tests were used to compare means of physical fitness test results of first-responders and healthcare providers, parity status (nulliparous [n = 27] vs. parous [n = 30]), who do (n = 30) and do not (n = 27) use hormonal birth control, and regular (n = 29) or irregular (n = 28) menstrual cycle. HCP = healthcare provider, BC = birth control, RSI = repetitive strain injury. RM = repetition maximum, R% = (absolute weight lifted in 4RM relative to bodyweight), Rt = right, L = left, reps = repetitions. *Significant two-sided p-value <0.05.
