# Supplementary material for: Musculoskeletal Injuries, Exercise Behaviors, and Reproductive Health Are Related to Physical Fitness of Female First-Responders and Health Care Providers
Source: Womens Health Rep (New Rochelle). 2024 May 3;5(1):393–403. doi: 10.1089/whr.2023.0189 (PMC11257141; doi:10.1089/whr.2023.0189)
Supplement: Supplementary Appendix C [file whr.2023.0189_suppl_appc.docx]

SDC 3.

Appendix C. Musculoskeletal injuries stratified by female reproductive health factors and occupation.

| Injury | Parity Status | | | Hormone Birth Control | | | Menstrual Cycle | | | Occupation | | |
| --- | --- | --- | --- | --- | --- | --- | --- | --- | --- | --- | --- | --- |
|  | Nulliparous % (n = 33) | Parous % (n = 30) | Significance | Yes %  (n = 30) | No %  (n = 27) | Significance | Irregular %  (n = 29) | Regular %  (n = 28) | Significance | FR %  (n = 19) | HCP %  (n = 38) | Significance |
| Acute | 74.1 | 80.0 | 0.594 | 70.0 | 85.2 | 0.172 | 71.4 | 82.8 | 0.308 | 78.9 | 76.3 | 0.823 |
| RSI | 74.1 | 83.3 | 0.392 | 76.7 | 81.5 | 0.656 | 75.0 | 82.8 | 0.473 | 94.7 | 71.1 | 0.045^ |
| Head, neck, shoulder | 44.4 | 53.3 | 0.503 | 43.3 | 55.6 | 0.357 | 42.9 | 55.2 | 0.352 | 68.4 | 39.5 | 0.039* |
| Upper extremity | 37.0 | 56.7 | 0.138 | 36.7 | 59.3 | 0.088 | 39.3 | 55.2 | 0.230 | 57.9 | 42.1 | 0.260 |
| Back | 44.4 | 46.7 | 0.866 | 40.0 | 51.9 | 0.370 | 42.9 | 48.3 | 0.681 | 78.9 | 28.9 | <0.001* |
| LPHC | 51.9 | 50.0 | 0.889 | 43.3 | 59.3 | 0.230 | 53.6 | 48.3 | 0.689 | 84.2 | 34.2 | <0.001* |
| Lower extermity | 63.0 | 73.3 | 0.400 | 63.3 | 74.1 | 0.384 | 64.3 | 72.4 | 0.509 | 68.4 | 68.4 | 1.000 |
| Pelvis | 7.4 | 10.0 | 1.000^ | 10.0 | 7.4 | 1.000^ | 10.7 | 6.9 | 0.670^ | 15.8 | 5.3 | 0.321 |
| Thorax/Ribs | 3.7 | 3.3 | 1.000^ | 3.3 | 3.7 | 1.000^ | 3.6 | 3.4 | 1.000^ | 5.3 | 2.6 | 1.000^ |
| Breast | 0.0 | 10.0 | 0.239^ | 3.3 | 7.4 | 0.599^ | 0.0 | 10.3 | 0.237^ | 15.8 | 0.0 | 0.033^* |
| Abdomen | 3.7 | 6.7 | 1.000^ | 6.7 | 3.7 | 1.000^ | 0.0 | 10.3 | 0.237^ | 15.8 | 0.0 | 0.033^* |
| Lower back | 40.7 | 40.0 | 0.955 | 40.0 | 40.7 | 0.955 | 42.9 | 37.9 | 0.705 | 78.9 | 21.1 | <0.001* |
| Upper back | 14.8 | 20.0 | 0.734^ | 13.3 | 22.2 | 0.492^ | 14.3 | 20.7 | 0.730^ | 21.1 | 15.8 | 0.717^ |
| Toes | 0.0 | 13.3 | 0.114^ | 6.7 | 7.4 | 1.000^ | 3.6 | 10.3 | 0.611^ | 5.3 | 7.9 | 1.000^ |
| Foot | 3.7 | 26.7 | **0.027*^** | 13.3 | 18.5 | 0.722^ | 7.1 | 24.1 | 0.144^ | 21.1 | 13.2 | 0.463^ |
| Ankle | 25.9 | 43.3 | 0.169 | 33.3 | 37.0 | 0.770 | 28.6 | 41.1 | 0.311 | 42.1 | 31.6 | 0.432 |
| Lower leg | 10.0 | 14.8 | 0.697 | 6.7 | 18.5 | 0.238^ | 14.3 | 10.3 | 0.706^ | 10.5 | 13.2 | 1.000 |
| Knee | 33.3 | 43.3 | 0.439 | 36.7 | 40.7 | 0.752 | 39.3 | 37.9 | 0.916 | 57.9 | 28.9 | 0.034* |
| Thigh | 7.4 | 3.3 | 0.599 | 6.7 | 3.7 | 1.000^ | 7.1 | 3.4 | 0.611^ | 15.8 | 0.0 | 0.033^* |
| Hip | 18.5 | 30.0 | 0.315 | 13.3 | 37.0 | 0.038* | 21.4 | 27.6 | 0.589 | 42.1 | 15.8 | 0.049^* |
| Fingers | 11.1 | 13.3 | 1.000 | 6.7 | 18.5 | 0.238 | 14.3 | 10.3 | 0.706 | 26.3 | 5.3 | 0.035^* |
| Thumb | 7.4 | 23.2 | 0.149^ | 16.7 | 14.8 | 1.000^ | 10.7 | 20.7 | 0.470^ | 10.5 | 18.4 | 0.703^ |
| Hand | 3.7 | 10.0 | 0.613^ | 0.0 | 14.8 | 0.044* | 7.1 | 6.9 | 1.000^ | 10.5 | 5.3 | 0.594^ |
| Wrist | 22.2 | 16.7 | 0.596 | 23.3 | 14.8 | 0.416 | 25.0 | 13.8 | 0.284 | 10.5 | 23.7 | 0.304^ |
| Lower arm | 11.1 | 6.7 | 0.660^ | 3.3 | 14.8 | 0.179^ | 3.6 | 13.8 | 0.352^ | 10.5 | 7.9 | 1.000^ |
| Elbow | 18.5 | 13.3 | 0.722^ | 20.0 | 11.1 | 0.476^ | 21.4 | 10.3 | 0.297^ | 26.3 | 10.5 | 0.143^ |
| Upper arm | 0.0 | 6.7 | 0.429^ | 0.0 | 7.4 | 0.220^ | 0.0 | 6.9 | 0.491^ | 5.3 | 2.6 | 1.000^ |
| Shoulder | 29.6 | 43.3 | 0.284 | 26.7 | 48.1 | 0.093 | 32.1 | 41.4 | 0.470 | 52.6 | 28.9 | 0.081 |
| Neck | 14.8 | 23.3 | 0.416 | 23.3 | 14.8 | 0.416 | 14.3 | 24.1 | 0.346 | 42.1 | 7.9 | 0.004^ |
| Head | 14.8 | 16.7 | 1.000^ | 13.3 | 18.5 | 0.722^ | 17.9 | 13.8 | 0.730^ | 26.3 | 10.5 | 0.143^ |

Chi-square analysis of musculoskeletal injuries by parity status (nulliparous [n = 27] vs. parous [n = 30]), participants who do (n = 30) and do not (n = 27) use hormonal birth control, regular (n = 29) and irregular (n = 28) menstrual cycle, and occupation (first-responder [n = 19] and healthcare provider [n = 38]). BC = birth control, RSI = repetitive strain injury, FR = first=responder, HCP = healthcare provider. ^ Fisher’s Exact (2-sided). *Significant difference p-value <0.05.
