# Supplementary material for: Musculoskeletal Injuries, Exercise Behaviors, and Reproductive Health Are Related to Physical Fitness of Female First-Responders and Health Care Providers
Source: Womens Health Rep (New Rochelle). 2024 May 3;5(1):393–403. doi: 10.1089/whr.2023.0189 (PMC11257141; doi:10.1089/whr.2023.0189)
Supplement: Supplementary Appendix D [file whr.2023.0189_suppl_appd.docx]

|  | Head, Neck, Shoulder Complex | | | Upper Extremity | | | Back | | | Lumbopelvic Hip Complex | | | Lower Extremity | | | |
| --- | --- | --- | --- | --- | --- | --- | --- | --- | --- | --- | --- | --- | --- | --- | --- | --- |
| Fitness Metric | Yes %  (n =28) | No %  (n = 29) | P-value | Yes %  (n = 27) | No %  (n = 39) | P-value | Yes %  (n = 26) | No %  (n = 31) | P-value | Yes %  (n = 29) | No %  (n = 28) | P-value | Yes %  (n = 39) | No %  (n = 18) | P-value |  |
| Sit-and-Reach (cm) | 38.1 ±7.9 | 35.2±6.9 | 0.143 | 36.0 ±8.6 | 37.2 ±6.4 | 0.558 | 38.2 ±6.7 | 35.4 ±8.0 | 0.164 | 38.5 ± 6.9 | 34.7 ±7.6 | 0.056 | 36.8 ±7.4 | 36.4 ± 8.0 | 0.841 |  |
| Long Jump (cm) | 162.1±25.0 | 156.2 ±25.9 | 0.382 | 156.9 ±22.9 | 161.6 ±27.7 | 0.491 | 159.6 ±25.2 | 158.6 ±26.0 | 0.882 | 157.6 ±27.3 | 160.6 ±23.7 | 0.668 | 156.6 ±28.0 | 164.4 ± 18.1 | 0.285 |  |
| Medicine Ball Toss (cm) | 270.1 ±57.6 | 252.8 ±47.5 | 0.219 | 269.2 ±58.6 | 254.1 ±47.1 | 0.286 | 286.2 ±46.9 | 240.4 ±49.1 | <0.001* | 274.1 ±56.1 | 248.0 ±46.7 | 0.062 | 260.8 ±55.2 | 262.4 ±49.3 | 0.917 |  |
| 4RM Back Squat (R%) | 118.3 ±30.4 | 126.8 ±35.8 | 0.340 | 118.4% ±29.0% | 126.5% ±36.8% | 0.367 | 120.9% ±38.8% | 124.1% ±28.4% | 0.732 | 116.8% ±33.9% | 128.7% ±32.1% | 0.180 | 118.0%±30.9% | 132.7%±36.8% | 0.121 |  |
| 4RM Back Squat (lbs) | 185.0 ±51.7 | 183.7 ±46.9 | 0.923 | 186.11 ±48.2 | 182.8 ±50.5 | 0.800 | 189.5 ± 59.4 | 180.0 ±38.7 | 0.486 | 182.1 ±54.0 | 186.7 ±44.1 | 0.724 | 176.7 ±48.3 | 201.0 ±47.7 | 0.081 |  |
| 4RM Bench Press (R%) | 63.4 ±18.4 | 62.3 ±18.4 | 0.822 | 61.2% ±16.3% | 64.3% ±19.9% | 0.535 | 61.4% ±17.7% | 64.0% ±18.9% | 0.593 | 61.7% ±18.1% | 64.0% ±18.6% | 0.629 | 61.5% ±17.2% | 65.6% ±20.5% | 0.436 |  |
| 4RM Bench Press (lbs) | 98.2 ±26.4 | 90.3 ±24.7 | 0.250 | 95.4 ±21.8 | 93.2 ±29.0 | 0.749 | 96.2 ±26.3 | 92.6 ±25.4 | 0.605 | 95.9 ±27.8 | 92.5 ±23.5 | 0.625 | 98.9 ±26.4 | 92.1 ±25.3 | 0.354 |  |
| Biering Sorenson (sec) | 163.8 ±68.1 | 169.8 ±52.5 | 0.711 | 161.5 ±69.3 | 171.6 ±51.4 | 0.532 | 152.3 ±52.2 | 179.0 ±64.5 | 0.096 | 151.3 ±48.9 | 182.9 ±67.1 | 0.047* | 172.7 ±61.2 | 154.2 ±57.6 | 0.286 |  |
| Single-leg Wall Sit (Rt) | 67.2 ±36.5 | 79.9 ±49.6 | 0.282 | 67.7 ±38.2 | 79.0 ±48.2 | 0.338 | 83.0 ±51.1 | 66.3±36.3 | 0.160 | 77.0 ±35.5 | 70.5 ±51.3 | 0.580 | 69.7 ±32.3 | 82.3 ±61.9 | 0.319 |  |
| Single-leg Wall Sit (L) | 65.6 ±33.5 | 73.2 ±48.3 | 0.501 | 64.3 ±33.4 | 74.0 ±47.8 | 0.390 | 75.7 ±36.4 | 64.6 ±45.4 | 0.324 | 72.5 ±31.2 | 66.5 ±50.4 | 0.854 | 68.8 ±42.8 | 71.0 ±40.3 | 0.425 |  |
| Push-ups (reps) | 25.0 ±13.0 | 23.41 ±11.3 | 0.625 | 23.4 ±10.9 | 24.9 ±13.2 | 0.662 | 22.3 ±10.8 | 25.8 ±13.1 | 0.285 | 22.6 ±12.2 | 25.86 ±12.0 | 0.601 | 24.8 ±12.4 | 22.9 ±11.6 | 0.854 |  |
| VO_2max_(mL/kg/min) | 41.1 ±7.0 | 44.5 ±7.5 | 0.094 | 41.1 ±7.4 | 44.3 ±7.3 | 0.118 | 41.3 ±7.4 | 44.0 ±7.4 | 0.191 | 42.1 ±7.2 | 43.6 ±7.7 | 0.684 | 42.6 ±6.5 | 43.5 ±9.2 | 0.601 |  |

SDC 4.

Appendix D. Physical fitness results by musculoskeletal injury stratified by body region

Note: T-tests were used to compare means of physical fitness test results of who have and have not sustained musculoskeletal injuries at the head, neck, shoulder complex, upper extremity (fingers, thumb, hand, wrist, lower arm, elbow upper arm, shoulder), back (neck, upper back, low back), lumbopelvic hip complex (low back, pelvis, hip), lower extremity (hip, thigh, knee, lower leg, ankle, foot, toes). RM = repetition maximum, R% = (bodyweight / absolute weight lifted in RM), Rt = right, L = left, reps = repetitions. *Significant two-sided p-value <0.05.
