# Supplementary material for: Musculoskeletal Injuries, Exercise Behaviors, and Reproductive Health Are Related to Physical Fitness of Female First-Responders and Health Care Providers
Source: Womens Health Rep (New Rochelle). 2024 May 3;5(1):393–403. doi: 10.1089/whr.2023.0189 (PMC11257141; doi:10.1089/whr.2023.0189)
Supplement: Supplementary Appendix E [file whr.2023.0189_suppl_appe.docx]

**SDC 5.**

**Appendix E. Physical fitness results by exercise behaviour**

|  |  | Physical training program by professional | | | | In-person fitness classes | | | | Virtual fitness classes | | | | | Team sports | | | | | Non-team sports | | | | | |
| --- | --- | --- | --- | --- | --- | --- | --- | --- | --- | --- | --- | --- | --- | --- | --- | --- | --- | --- | --- | --- | --- | --- | --- | --- | --- |
|  |  | N | Mean | Std. Deviation | p-value | N | Mean | Std. Deviation | p-value | | N | Mean | Std. Deviation | p-value | | N | Mean | Std. Deviation | p-value | | N | Mean | Std. Deviation | p-value |  |
| Sit and reach (cm) | Yes | 25 | 39.96 | 5.35 | <0.001* | 21 | 38.71 | 8.23 | 0.114 | | 20 | 33.58 | 7.75 | 0.021* | | 22 | 35.46 | 6.25 | 0.218 | | 21 | 37.98 | 7.06 | 0.315 |  |
|  | No | 31 | 33.89 | 8.03 |  | 36 | 35.46 | 6.86 |  | | 37 | 38.32 | 6.89 |  | | 34 | 37.91 | 7.74 |  | | 36 | 35.89 | 7.73 |  |  |
| Long jump (cm) | Yes | 25 | 163.38 | 22.44 | 0.180 | 21 | 165.34 | 25.03 | 0.158 | | 20 | 145.45 | 18.76 | 0.002* | | 22 | 169.87 | 23.47 | 0.012* | | 21 | 162.04 | 27.15 | 0.507 |  |
|  | No | 31 | 154.31 | 26.63 |  | 36 | 155.44 | 25.26 |  | | 37 | 166.45 | 25.68 |  | | 34 | 152.48 | 24.86 |  | | 36 | 157.36 | 24.57 |  |  |
| Medicine ball toss (cm) | Yes | 25 | 269.91 | 38.42 | 0.217 | 21 | 263.62 | 47.39 | 0.801 | | 20 | 236.70 | 55.38 | 0.009* | | 22 | 278.09 | 52.81 | 0.068 | | 21 | 273.70 | 49.87 | 0.178 |  |
|  | No | 31 | 252.95 | 62.14 |  | 36 | 259.91 | 56.56 |  | | 37 | 274.56 | 47.19 |  | | 34 | 251.56 | 51.49 |  | | 36 | 254.03 | 54.04 |  |  |
| Back Squat 4RM (absolute)(kg) | Yes | 25 | 203.52 | 50.64 | 0.005* | 21 | 183.57 | 43.16 | 0.928 | | 20 | 169.50 | 45.82 | 0.093 | | 22 | 199.68 | 58.14 | 0.073 | | 21 | 198.24 | 57.41 | 0.103 |  |
|  | No | 31 | 167.58 | 42.19 |  | 36 | 184.81 | 52.71 |  | | 37 | 192.38 | 49.39 |  | | 34 | 175.59 | 40.52 |  | | 36 | 176.25 | 42.15 |  |  |
| Back Squat 4RM (R%) | Yes | 25 | 1.35 | 0.34 | 0.009* | 21 | 1.27 | 0.30 | 0.487 | | 20 | 1.13 | 0.28 | 0.118 | | 22 | 1.29 | 0.37 | 0.286 | | 21 | 1.29 | 0.38 | 0.247 |  |
|  | No | 31 | 1.12 | 0.30 |  | 36 | 1.20 | 0.35 |  | | 37 | 1.28 | 0.35 |  | | 34 | 1.19 | 0.31 |  | | 36 | 1.19 | 0.30 |  |  |
| Bench Press 4RM absolute (kg) | Yes | 25 | 107.20 | 22.55 | <0.001* | 21 | 95.00 | 18.44 | 0.861 | | 20 | 86.00 | 21.00 | 0.075 | | 22 | 101.59 | 25.79 | 0.100 | | 21 | 97.86 | 29.35 | 0.417 |  |
|  | No | 31 | 83.71 | 23.77 |  | 36 | 93.75 | 29.26 |  | | 37 | 98.65 | 27.05 |  | | 34 | 90.00 | 25.02 |  | | 36 | 92.08 | 23.37 |  |  |
| Bench press 4RM (R%) | Yes | 25 | 0.71 | 0.17 | 0.002* | 21 | 0.66 | 0.13 | 0.385 | | 20 | 0.58 | 0.14 | 0.117 | | 22 | 0.65 | 0.17 | 0.393 | | 21 | 0.64 | 0.20 | 0.724 |  |
|  | No | 31 | 0.56 | 0.17 |  | 36 | 0.61 | 0.21 |  | | 37 | 0.66 | 0.20 |  | | 34 | 0.61 | 0.20 |  | | 36 | 0.62 | 0.18 |  |  |
| Sorensen (seconds) | Yes | 25 | 171.46 | 56.42 | 0.589 | 21 | 175.66 | 51.86 | 0.402 | | 20 | 156.89 | 74.42 | 0.419 | | 22 | 167.45 | 59.14 | 0.798 | | 21 | 183.73 | 57.02 | 0.106 |  |
|  | No | 31 | 162.54 | 64.60 |  | 36 | 161.66 | 64.71 |  | | 37 | 172.18 | 51.28 |  | | 34 | 163.27 | 59.71 |  | | 36 | 156.95 | 60.57 |  |  |
| Single leg wall sit (Rt) (cm) | Yes | 25 | 84.91 | 54.48 | 0.125 | 21 | 76.80 | 39.11 | 0.690 | | 20 | 68.16 | 41.45 | 0.483 | | 21 | 78.89 | 51.48 | 0.544 | | 21 | 78.13 | 49.84 | 0.567 |  |
|  | No | 30 | 65.59 | 31.23 |  | 35 | 71.91 | 46.94 |  | | 36 | 76.84 | 45.42 |  | | 34 | 71.37 | 39.43 |  | | 35 | 71.11 | 40.40 |  |  |
| Single leg wall sit (L) (cm) | Yes | 25 | 71.52 | 38.55 | 0.813 | 21 | 72.02 | 35.56 | 0.731 | | 20 | 66.81 | 54.01 | 0.720 | | 21 | 68.09 | 31.17 | 0.766 | | 21 | 75.44 | 35.19 | 0.415 |  |
|  | No | 30 | 68.80 | 44.98 |  | 35 | 68.02 | 45.30 |  | | 36 | 71.03 | 33.64 |  | | 34 | 71.57 | 47.39 |  | | 35 | 65.97 | 45.16 |  |  |
| Push-up (reps) | Yes | 25 | 26.44 | 9.72 | 0.197 | 21 | 25.67 | 8.21 | 0.487 | | 20 | 20.75 | 10.03 | 0.115 | | 22 | 25.00 | 8.87 | 0.721 | | 21 | 26.76 | 13.94 | 0.224 |  |
|  | No | 31 | 22.19 | 13.71 |  | 36 | 23.33 | 13.91 |  | | 37 | 26.05 | 12.82 |  | | 34 | 23.79 | 14.04 |  | | 36 | 22.69 | 10.81 |  |  |
| VO2max(mL/kg/min) | Yes | 23 | 44.59 | 6.70 | 0.129 | 20 | 44.08 | 7.04 | 0.358 | | 20 | 41.25 | 8.12 | 0.221 | | 19 | 44.91 | 7.25 | 0.132 | | 19 | 43.36 | 6.96 | 0.721 |  |
|  | No | 29 | 41.41 | 7.89 |  |  | 42.13 | 7.66 |  | | 33 | 43.84 | 6.91 |  | | 33 | 41.64 | 7.48 |  | | 34 | 42.59 | 7.76 |  |  |

**Note: T-tests were used to compare means of physical fitness test results of those who participated in physical training program created by a professional, in-person fitness classes, virtual fitness classes, team sports, or non-team sports within the last year. RM = repetition maximum, R% = (bodyweight / absolute weight lifted in RM), Rt = right, L = left, reps = repetitions. *Significant two-sided p-value <0.05.**
